# Exploring relationships between autistic traits and body temperature, circadian rhythms, and age

**DOI:** 10.1038/s41598-023-32449-z

**Published:** 2023-04-11

**Authors:** Souta Hidaka, Mizuho Gotoh, Shinya Yamamoto, Makoto Wada

**Affiliations:** 1 Department of Psychology, Rikkyo University, 1-2-26, Kitano, Niiza, Saitama, 352-8558 Japan; 2 Integrative Neuroscience Research Group, Human Informatics and Interaction Research Institute, National Institute of Advanced Industrial Science and Technology (AIST), 1-1-1, Umezono, Tsukuba, 305-8568 Japan; 3 Graduate School of Comprehensive Human Sciences, University of Tsukuba, 1-1-1, Tennodai, Tsukuba, 305-8577 Japan; 4 Developmental Disorders Section, Department of Rehabilitation for Brain Functions, Research Institute of National Rehabilitation Center for Persons with Disabilities, 4-1, Namiki, Tokorozawa, Saitama, 359-8555 Japan; 5 Department of Psychology, Faculty of Human Sciences, Sophia University, 7-1, Kioi-cho, Chiyoda-ku Tokyo, 102-8554 Japan

**Keywords:** Psychology, Human behaviour

## Abstract

The number of clinical diagnoses of autism spectrum disorder (ASD) is increasing annually. Interestingly, the human body temperature has also been reported to gradually decrease over the decades. An imbalance in the activation of the excitatory and inhibitory neurons is assumed to be involved in the pathogenesis of ASD. Neurophysiological evidence showed that brain activity decreases as cortical temperature increases, suggesting that an increase in brain temperature enhances the inhibitory neural mechanisms. Behavioral characteristics specific to clinical ASD were observed to be moderated when people with the diagnoses had a fever. To explore the possible relationship between ASD and body temperature in the general population, we conducted a survey study using a large population-based sample (N ~ 2000, in the age groups 20s to 70s). Through two surveys, multiple regression analyses did not show significant relationships between axillary temperatures and autistic traits measured by questionnaires (Autism Spectrum (AQ) and Empathy/Systemizing Quotients), controlling for covariates of age and self-reported circadian rhythms. Conversely, we consistently observed a negative relationship between AQ and age. People with higher AQ scores tended to have stronger eveningness. Our findings contribute to the understanding of age-related malleability and the irregularity of circadian rhythms related to autistic traits.

## Introduction

Autism spectrum disorder (ASD) is a neurodevelopmental disorder with typical symptoms including deficits in social communication and interactions, and irregularities in behavioral aspects, such as restricted and repetitive interests^1^. The current understanding of ASD is that ASD-like characteristics are not unique to those with clinical ASD diagnoses, but rather common properties (autistic traits) falling on a continuous distribution among the general population^2–6^. The number of clinical ASD diagnoses is reported to be increasing annually^7^. While explanations such as changes in diagnostic criteria and social circumstances are consistent with the recent spectrum view of ASD, these factors cannot fully explain the increase in the number of diagnoses^7^.

It has been suggested that an imbalance in the activation of excitatory and inhibitory neurons in the brain could be involved in the pathogenesis of ASD^8,9^. Specifically, characteristics related to ASD are assumed to increase with relative increases/decreases in excitatory/inhibitory neuronal activities, respectively. It has been reported that a shift of the excitatory-inhibitory balance toward the excitatory side resulted in an ASD-like phenotype in mice^8^. Notably, neurophysiological evidence shows that, in some brain areas, brain activity increases as cortical temperature decreases^10–15^. Pharmacological evidence further suggests that a decrease in cortical temperature weakens the contribution of inhibitory inputs to neural activity relative to excitatory inputs^15^. These findings lead to an assumption that lower brain temperature is associated with higher autistic traits because of the excitatory-dominant neuronal activities. This assumption is supported by the following evidence. First, human body temperature has been reported to be gradually declining worldwide^16^. Given that brain temperature declines along with body temperature, the excitatory-inhibitory balance in the brain is assumed to be biased toward the excitatory side. This may result in the increase of ASD-like characteristics and the number of clinical ASD diagnoses at a population level. Second, the frequency and severity of behavioral characteristics specific to ASD were moderated in children with clinical ASD diagnoses when they had a fever^17^. This could be interpreted to mean that, with an elevation of body temperature, the inhibitory nervous system becomes dominant, resulting in a temporary decrease in behaviors related to ASD.

Development of questionnaires and surveys using these questionnaires has supported the spectrum view of autistic traits^2–6^. The autism-spectrum quotient (AQ) is a self-reported questionnaire developed as a measure to assess autistic traits among individuals both with and without the diagnosis^4^. The scores are demonstrated to be distributed with higher scores indicating a greater magnitude of autistic traits, as people with clinical ASD diagnosis showed larger AQ values^4,6^. Different questionnaires have also been developed to measure empathizing (Empathy Quotient; EQ) and systemizing (Systemizing Quotient; SQ) aspects related to ASD^18–21^. While AQ is designed to estimate general tendencies related to ASD, these questionnaires characterize social and behavioral aspects particular to ASD in two dimensions: empathizing is defined as “the drive to identify another person’s emotions and thoughts, and to respond to these with an appropriate emotion”, and systemizing is defined as “the drive to analyze, understand, predict, control, and construct rule-based systems”^18^. It has been shown that AQ scores are negatively and positively related to EQ and SQ scores, respectively^18^, and that there is a negative relationship between EQ and SQ^18,19^. These scores are also shown to be distributed, and smaller and larger scores of EQ and SQ, respectively, have been observed for people both with and without a clinical ASD diagnosis^18,22^. Furthermore, consistent with the fact that males showed a higher rate of clinical ASD diagnosis^23^, previous studies reported higher AQ and SQ scores and lower EQ scores for males compared to females^4,18,19,24^. The evidence regarding AQ, EQ, and SQ is confirmed to be cross-cultural; for example, findings obtained in the United Kingdom have been replicated in the Japanese population^19,22,24^. These findings suggest that ASD-like characteristics are common properties falling on a continuous distribution among the general population, and AQ, EQ, and SQ are reliable, universal measurements of autistic traits.

The above-mentioned findings posited an idea of a possible relationship between autistic traits and normal body temperature in a general population in view of the ASD spectrum; relatively lower body temperatures might be observed for people with higher autistic traits if the decrease in body temperature is associated with the general increase in clinical ASD diagnosis based on an imbalance of activation in excitatory and inhibitory neural circuits in the brain. We tested this possibility by performing a large-scale questionnaire survey (N ~ 2000). We measured autistic traits by using AQ, EQ and SQ. As for body temperature, we asked the participants to report their axillary temperatures. Circadian rhythm (morningness/eveningness), age^25^, and sex^26^ have been reported to be modulatory factors. In addition to collecting data on age and sex as control variables, we asked participants to answer a questionnaire (Composite Morning Questionnaire; CSM)^27,28^ as an index of the circadian rhythm.

It should be noted that people with clinical ASD diagnosis are reported to have circadian rhythm disturbances^29^ along with sleep problems^30^. However, to the best of our knowledge, no study has investigated the relationship between autistic traits and circadian rhythms in a large sample of the general population. Thus, we explored the relationship between autistic traits and circadian rhythm. Regarding the relationship between autistic traits and age, a study performed in the United States reported that there existed a negative relationship between AQ scores and age^31^. We explored the relationships between age and AQ, EQ, and SQ scores in the Japanese population.

We conducted an online survey using a large population-based Japanese sample in the age groups 20s to 70s and an equal distribution of sexes. As for a possible relationship between autistic traits and body temperature, we predicted that people with greater autistic traits, that is, higher AQ and SQ scores and lower EQ scores, would have lower body temperatures based on the evidence for the longitudinal shifts in clinical ASD diagnosis^7^ and body temperatures^16^. We also expected that negative relationships^31^ between autistic traits and age. We did not have any hypotheses regarding the relationship between autistic traits and circadian rhythms. We conducted the surveys twice at different time points to check the reproducibility and validity of our findings.

## Methods

### Preregistrations

We preregistered our research protocols on the Open Science Framework (the first survey (Survey 1): https://osf.io/z5dmj; the second survey (Survey 2): https://osf.io/268na). We made some modifications after the data acquisition in the first survey. These changes are described in this section and are retained for the second survey.

### Participants

We designed our data collection such that the data of every 400 participants were assigned to either the 20s, 30s, 40s, 50s, 60s, or 70s age group, and sex was equally distributed across the age groups (2400 participants’ data in total). In the Surveys 1/2, we collected the data of 3227/3402 participants using the database and platform of an online survey company (Cross Marketing, Japan), but excluded those of 827/1020 participants from analyses since their strategy was considered inappropriate (“Satisfice”) by a filler question (Supplementary Fig. 1). We also excluded data using previously determined methods (see “Data exclusions” section for details). Consequently, the analyzed data consisted of 2185/2264 participants for analyses related to body temperature and 2211/2288 participants for other analyses in Surveys 1/2. Each age group and sex was almost equally distributed. Surveys 1/2 were conducted from October 22 to 27, 2021/January 14 to 19, 2022, in Japan. The study was approved by the Ethics Committee of the Department of Psychology, Rikkyo University (Reference number: 21–30), and was conducted according to the approved guidelines and the Declaration of Helsinki. Informed consent was obtained from each participant by asking them to check the “yes” mark if they agreed to participate in the survey.

### Questionnaires

We gave the following instructions to the participants (excerpted from the section related to the study questionnaires): “This survey is conducted to investigate whether there is a relationship between people’s body temperature and thinking, behavior, and lifestyle patterns. We will ask you questions about your body temperature and things related to your daily life”. Demographic information included age, sex (male, female, do not know/do not want to answer), and state of residence were collected (Supplementary Fig. 1). We confirmed that our data comprised people from all areas (47 prefectures) across Japan in both surveys.

We asked the participants to report their body temperatures measured using an axillary thermometer (values to one decimal place). We also asked them to report the time (hours and minutes) when body temperature was measured and when the participants woke up on the participating day (hours and minutes). In Survey 1, the manufacturer and model number of the thermometer were reported if the participants knew. In Survey 2, we asked the participants to confirm that they measured body temperature with an axillary thermometer through the question, “Did you measure your body temperature using an axillary thermometer?”.

Participants were asked to report their physical condition as related to the body temperature by the following question: “Please tell us if there is anything in your current physical condition that affects your body temperature; not applicable, symptoms of a cold, immediately after exercise, medication with antipyretic effect (antipyretic analgesic), vaccination against new coronaviruses within 1 week, other (free description)”. While the menstrual cycle was considered to affect body temperature in females, we did not explicitly ask to report that because of its large inter-day as well as inter-person variability^32,33^. Some participants voluntarily reported menstruation as a factor affecting body temperature, and we excluded their data from the analyses.

For the measurements of autistic traits, we used the Japanese version of the AQ (50 items)^24^ and the Japanese version of the short form of EQ-SQ (47 items)^19,22^. The circadian rhythm was evaluated as morningness/eveningness using the Japanese version of the CSM (13 items)^27^. In each series of AQ and EQ-SQ question, we included the item “please check the right/left-most answer in this question” as the filler question to detect the use of an inappropriate strategy (“Satisfice” i.e., the strategy to fill the left-/right-most marks for all items without reading them).

### Data exclusions

We performed data exclusion as follows, after excluding participants whose strategy was regarded as inappropriate (“Satisfice”) by the filler question. First, we excluded the data if answers other than “not applicable” were reported in the question regarding the physical condition related to body temperature. In our preregistered protocol, we planned to exclude participants if AQ, EQ, SQ, CSM, body temperature, and time index (time of body temperature measurement − time of waking up) exceed ± 2 SD. However, after the data collection of Survey 1, we modified these data exclusion methods. As for body temperature, the criterion of ± 2 SD resulted in the exclusion of data that could be regarded as within a normal range. Thus, we used the criterion of ± 3 SD. As for AQ, EQ-SQ, and CSM, we realized that it should be reasonable for people with diagnostic levels to show irregularly higher values in the general population. Therefore, an outlier exclusion was not performed. The time data were excluded if they exceeded 24 h because the time index was not normally distributed and was heavily skewed. We also excluded two participants whose body temperatures were not measured using an axillary thermometer based on the reported manufacturer and model.

### Data analyses

We calculated AQ, EQ, SQ, and CSM scores based on the calculation method defined in previous studies. Higher AQ and SQ scores and lower EQ scores indicate stronger autistic traits. Higher CSM scores indicate greater morningness/weaker eveningness. Diurnal variations were reported to occur in body temperature from waking up to sleep^25^. Thus, we calculated the time index by subtracting the reported time of waking up from the body temperature. The time of body temperature measurement and that of waking up were converted to minutes starting from 0 a.m (the beginning of a day).

For the main analysis, we performed hierarchical multiple linear regression analysis using the forced entry method. First, multiple regression analysis was conducted with body temperature as the response variable and the following explanatory variables: age, CSM, time, age × CSM, age × time, and CSM × time. Age, CSM, and the time index were centered. By including factors possibly related to body temperature, we checked whether and how these factors affect the obtained body temperature data and controlled for possible confounding effects other than autistic traits. Second, we further entered the AQ, EQ, and SQ scores as explanatory variables into the multiple linear regression model and tested whether the amount of regression significantly increased. This method also enabled us to control for positive or negative correlations between AQ, EQ, and SQ scores^18^. Females have been reported to have a higher body temperature than males^26^. It has also been reported that males have higher AQ and SQ scores and females have higher EQ scores^18^. To take into consideration these sex-related differences, we performed the above-mentioned hierarchical multiple linear regression analysis for each sex group. We confirmed the normality of the residuals based on Q–Q plots.

Additional analyses were performed, exploratory in Survey 1 and confirmatory in Survey 2. We performed a hierarchical multiple regression analysis using CSM as the response variable. In the first step, age was included as an explanatory variable (In our preregistrations, we report that the time index was entered in the first step. We noticed this as a typo because no relationship could be assumed between the time index and CSM). AQ, EQ, and SQ scores were then included in the model. We also tested whether the dependent variables differed between the sexes. The correlation coefficients were calculated for AQ, EQ, SQ, and age.

We also checked whether the reported body temperature values differed among thermometer manufacturers in the first survey. Originally, we intended to standardize the body temperature data if we found a significant main effect of the manufacturer of axillary thermometers or interaction effects in the analysis of variance (ANOVA) for body temperature with factors of the manufacturer and age group (20–70s). Age groups were included as an independent variable in this ANOVA because participants’ age was considered to affect the choice of manufacturer. Approximately 58% of participants provided information about manufacturers, and only three manufacturers, the reported number of which was over 100, were identified as available for ANOVA. Therefore, we did not standardize the temperature data of the specific manufacturers, and tested the possible effects by comparing the results with and without the specific manufacturers’ data. We confirmed the normality of the residuals based on Q–Q plots.

Before data collection, we performed a power analysis for a multiple linear regression using G*Power 3.1 software^34^ with an effect size of f^2^ = 0.15, alpha of 0.05, power of 0.8, and three predictors. This indicated that 77 participants were required. Our sample size was appropriately powered to detect comparably sized effects. Data analyses and visualizations were performed using the R software (version 4.0.3) with ggplot2^35^, psych^36^, dplyr^37^ and JASP (version 0.16)^38^. The alpha level for statistical tests was set at 0.05 using the Bonferroni correction. We also reported the Bayes factors.

## Results

### Survey 1

#### Analyses of body temperature

From the obtained data (Table 1), we performed a hierarchical multiple regression analysis for body temperature (Table 2). First, we included age, CSM, time, and their interaction terms as explanatory variables. With a significant model-fit (*F*(6, 2184) = 9.90, *p* < 0.001, adjusted R^2^ = 0.02, BF_10_ = 2.04 × 10^6^), we found a significant negative relationship for age (*β* =  − 0.16, *t* =  − 6.95, *p* < 0.001). Second, the inputs of AQ, EQ, and SQ scores did not significantly changes in the explanatory effect of the model (*F*(3, 2178) = 2.43, *p* = 0.06, R^2^ change = 0.003, BF_10_ = 0.11). We performed the same analysis for data of both male and female participants as planned. We found a significant change in the explanatory effect in the second step for the data of males (*F*(3, 1093) = 3.82, *p* = 0.01, R^2^ change = 0.01, BF_10_ = 2.12). This showed significant negative relationships between AQ and EQ (*β*s =  − 0.09, *ts* <  − 2.28, *ps* < 0.02). In contrast, there was no significant change in the explanatory effect for the data of females participants (*F*(3, 885) = 0.65, *p* = 0.58, R^2^ change = 0.002, BF_10_ = 0.02) (Supplementary Table 1).

**Table 1:** Descriptive statistics of data in Survey 1.

Label	N	Mean	SD	Minimum	Maximum
Body temperature	2185	36.27	0.31	35.30	37.20
AQ	2211	21.65	6.99	4.00	46.00
EQ	2211	14.85	7.67	0.00	43.00
SQ	2211	14.19	8.97	0.00	50.00
CSM	2211	37.43	7.99	13.00	55.00
Wakeup time	2211	436.27	141.44	0.00	1426.00
Measurement time	2211	791.67	321.02	0.00	1438.00
Time	2211	419.87	320.74	0.00	1439.00
Age	2211	49.94	16.45	20.00	79.00

**Table 2:** Results for the multiple regression analysis for body temperature in Survey 1.

Model	Variables	*β*	*t*	*p*	VIF
First step	(Intercept)		5061.75	< 0.001	
	Age	− 0.16	− 6.95	< 0.001	1.18
	CSM	0.00	0.12	0.91	1.19
	Time	0.02	0.71	0.48	1.01
	Age × CSM	0.01	0.67	0.50	1.01
	Age × time	− 0.02	− 0.98	0.33	1.17
	CSM × time	− 0.02	− 0.89	0.37	1.17
Second step	(Intercept)		949.11	< 0.001	
	Age	− 0.17	− 7.32	< 0.001	1.25
	CSM	0.00	0.00	1.00	1.22
	Time	0.02	0.76	0.45	1.01
	Age × CSM	0.01	0.56	0.58	1.02
	Age × time	− 0.02	− 0.99	0.32	1.17
	CSM × time	− 0.02	− 0.89	0.38	1.17
	AQ	− 0.06	− 2.31	0.02	1.61
	EQ	− 0.07	− 2.45	0.01	1.60
	SQ	0.01	0.37	0.71	1.09

To further investigate these differences between the sexes, we performed an ANOVA for body temperature with age group (20s to 70s) and sex (male/female). Besides the significant effect of age group (*F*(5, 2166) = 15.64, *p* < 0.001, BF_10_ = 3.43 × 10^11^) and no significant effect of sex (*F*(1, 2166) = 3.28, *p* = 0.07, BF_10_ = 0.20), we found a significant interaction (*F*(5, 2166) = 2.55, *p* = 0.03, BF_10_ = 0.09). Simple main effects showed that the body temperatures of females were significantly lower than those of males in the 60s and 70s age group (*Fs* > 3.88; *ps* < 0.05) (Supplementary Fig. 2), which might be due to menopause at these ages. We then performed a hierarchical multiple regression analysis for body temperatures excluding females’ 60s and 70s data, and found that the results were consistent with the male data; there were significant changes in the explanatory effect in the second step (*F*(3, 1798) = 3.86, *p* = 0.01, R^2^ change = 0.01, BF_10_ = 1.23) and significant negative relationships for AQ and EQ (*β*s <  − 0.07, *ts* <  − 2.36, *ps* < 0.02) (Supplementary Table 2).

We also checked whether the reported body temperatures differed among the thermometer manufacturers. We identified that the number was over 100 for three manufacturers and included four labels (three manufactures (1–3) and the others (0)) in an ANOVA. The ANOVA with factors manufacturing labels and age groups found a significant difference for the manufacturer label (*F*(3, 2161) = 6.42, *p* < 0.001, BF_10_ = 34.47) and age (*F*(5, 2161) = 8.80, *p* < 0.001, BF_10_ = 8.02 × 10^11^), while there was no significant interaction (*F*(15, 2161) = 0.81, *p* = 0.67, BF_10_ = 9.65 × 10^4^). Post hoc tests found that one company (label 2) had significantly higher values than the others (label 0) (*t* =  − 3.32, *p* = 0.005), and another company (label 3) had lower values than the other companies (labels 1 and 2) (*ts* > 2.80, *ps* < 0.03). We performed a hierarchical multiple regression analysis for body temperature by excluding these two companies’ data and confirmed that the results were consistent with those of the whole data (Supplementary Table 3).

#### Analyses for CSM

We also performed a hierarchical multiple regression analysis for the CSM score (Table 3). In the first step, we included age and found a significant model-fit (*F*(1, 2210) = 406.91, *p* < 0.001, adjusted R^2^ = 0.16, BF_10_ = 2.67 × 10^79^). There was a significant positive relationship for age (*β* = 0.39, *t* = 20.17, *p* < 0.001). The inputs of AQ, EQ, and SQ scores significantly changed the explanatory effect of the model (*F*(3, 2209) = 15.72, *p* < 0.001, R^2^ change = 0.02, BF_10_ = 2.46 × 10^10^), and showed a significant negative relationship for AQ (*β* =  − 0.12, *t* <  − 4.77, *p* < 0.001). These results were consistent for both male and female participants (Supplementary Table 4): The explanatory effect of the model changed significantly in the second step (Male; *F*(3, 1110) = 9.31, *p* < 0.001, R^2^ change = 0.02, BF_10_ = 479.13; Female; *F*(3, 1088) = 6.31, *p* < 0.001, R^2^ change = 0.01, BF_10_ = 6.94), and showed a significant negative relationship for AQ (Male: *β* =  − 0.12, *t* =  − 3.32, *p* < 0.001; Female: *β* =  − 0.11, *t* =  − 3.16, *p* = 0.001) as well as a significant negative relationship for SQ in male (*β* = 0.08, *t* = 2.47, *p* = 0.01).

**Table 3:** Results for the multiple regression analysis for CSM in Survey 1.

Model	Variables	*β*	*t*	*p*	VIF
First step	(Intercept)		55.86	< 0.001	
	Age	0.39	20.17	< 0.001	1.00
Second step	(Intercept)		27.76	< 0.001	
	Age	0.36	17.92	< 0.001	1.09
	AQ	− 0.12	− 4.77	< 0.001	1.60
	EQ	0.02	0.74	0.46	1.61
	SQ	0.03	1.65	0.10	1.09

#### Analyses for sex

We tested whether there were differences in the dependent variables (*p* = 0.05/8) between the sexes. Mann–Whitney U tests found that AQ and SQ scores were significantly higher for males than for females, and the opposite tendency was found for EQ (Table 4).

**Table 4:** Results for the comparisons between sexes in Survey 1.

Label	W	*p*	BF10
Body temperature	621,011.0	0.05	0.23
AQ	710,808.0	< 0.001	3.89E+06
EQ	494,246.5	< 0.001	1.57E+05
SQ	883,644.0	< 0.001	1.079E+18
CSM	591,141.5	0.28	0.12
Wakeup time	610,452.5	0.83	0.05
Measurement time	605,202.5	0.90	0.05
Time	616,549.0	0.53	0.07

#### Analyses for autistic traits

The relationships among autistic traits were also tested (*p* = 0.05/3). Correlation analyses with Kendall’s tau showed significant negative correlations between AQ and both EQ (*τ* =  − 0.43, *p* < 0.001, BF_10_ = 6.18 × 10^201^) and SQ (*τ* =  − 0.09, *p* < 0.001, BF_10_ = 3.42 × 10^7^). SQ and EQ were positively correlated (*τ* = 0.16, *p* < 0.001, BF_10_ = 1.86 × 10^27^). These results were consistent for both male and female participants (Supplementary Table 5).

We also performed partial correlation analyses for autistic traits against age (*p* = 0.05/3). There were significant negative relationships for AQ (*τ* =  − 0.17, *p* < 0.001) and EQ (*τ* =  − 0.05, *p* < 0.001) and a positive one for SQ (*τ* = 0.04, *p* = 0.008). A significant relationship for AQ score was consistently observed in both male and female participants, whereas significant relationships for EQ and SQ were found only in the males (Supplementary Table 6).

### Survey 2

We performed a second survey to check whether the findings of Survey 1 were reproduced at different time points. Since we confirmed that the main results were consistent with the differences in the thermometer manufacturers, we did not collect this information in Survey 2 and asked the participants to confirm that they measured their body temperature with an axillary thermometer. Except for this, the procedures used were identical to those used in Survey 1. Descriptive information is presented in Table 5.

**Table 5:** Descriptive statistics of data in Survey 2.

Label	N	Mean	SD	Minimum	Maximum
Body temperature	2264	36.25	0.33	35.20	37.20
AQ	2288	21.54	6.76	4.00	45.00
EQ	2288	14.89	7.55	0.00	44.00
SQ	2288	14.10	8.83	0.00	48.00
CSM	2288	37.20	7.82	13.00	55.00
Wakeup time	2288	457.85	142.74	0.00	1430.00
Measurement time	2288	829.10	314.46	3.00	1439.00
Time	2288	432.30	320.86	0.00	1439.00
Age	2288	49.52	16.53	20.00	79.00

#### Analyses of body temperature

The hierarchical multiple regression analysis for body temperature (Table 6) showed a significant model-fit in the first step (*F*(6, 2263) = 14.45, *p* < 0.001, adjusted R^2^ = 0.03, BF_10_ = 8.38 × 10^11^). This revealed a significant negative relationship with age (*β* =  − 0.18, *t* =  − 8.07, *p* < 0.001). The inputs of AQ, EQ, and SQ scores did not significantly change the explanatory effect in the model (*F*(3, 2257) = 2.13, *p* = 0.10, R^2^ change = 0.003, BF_10_ = 0.06). The same tendencies were observed in both male and female participants (male: *F*(6, 1138) = 2.69, *p* = 0.01, adjusted R^2^ = 0.01, BF_10_ = 0.01, with a significant effect of age (*β* =  − 0.10, *t* =  − 3.34, *p* = 0.002) in the first step and *F*(3, 1132) = 1.30, *p* = 0.27, R^2^ change = 0.003, BF_10_ = 0.06 in the second step; female: *F*(6, 1117) = 16.02, *p* < 0.001, adjusted R^2^ = 0.08, BF_10_ = 1.12 × 10^14^ with a significant effect of age (*β* =  − 0.25, *t* =  − 7.99, *p* < 0.001) in the first step and *F*(3, 1111) = 0.57, *p* = 0.64, R^2^ change = 0.001, BF_10_ = 0.01 in the second step) (Supplementary Table 7). The ANOVA with age group × sex showed a significant interaction (*F*(5, 2245) = 4.77, *p* < 0.001, BF_10_ = 18.37) as well as significant main effects of age group (*F*(5, 2245) = 19.55, *p* < 0.001, BF_10_ > 100) and sex (*F*(1, 2245) = 4.32, *p* = 0.04, BF_10_ = 4.49). In simple main effects, the body temperatures of females were significantly lower than those of males in the 50s and 70s age groups (*Fs* > 4.61; *ps* < 0.03) (Supplementary Fig. 3). As in Survey 1, we performed a hierarchical multiple regression analysis for body temperatures, excluding females’ 50s and 70s data. Consistent with the analyses of the entire data, this analysis did not find a significant change in the explanatory effect in the second step (*F*(3, 1876) = 2.03, *p* = 0.11, R^2^ change = 0.003, BF_10_ = 0.08) (Supplementary Table 8).

**Table 6:** Results for the multiple regression analysis for body temperature in Survey 2.

Model	Variables	*β*	*t*	*p*	VIF
First step	(Intercept)		4995.76	< 0.001	
	Age	− 0.18	− 8.07	< 0.001	1.15
	CSM	− 0.03	− 1.36	0.18	1.15
	Time	0.00	0.10	0.92	1.01
	Age × CSM	− 0.02	− 1.00	0.32	1.01
	Age × time	− 0.01	− 0.65	0.51	1.10
	CSM × time	0.01	0.66	0.51	1.10
Second step	(Intercept)		930.08	< 0.001	
	Age	− 0.19	− 8.32	< 0.001	1.23
	CSM	− 0.04	− 1.57	0.12	1.18
	Time	0.00	0.12	0.90	1.01
	Age × CSM	− 0.02	− 1.07	0.29	1.01
	Age × time	− 0.01	− 0.62	0.54	1.10
	CSM × time	0.02	0.73	0.47	1.10
	AQ	− 0.04	− 1.72	0.09	1.50
	EQ	0.04	1.97	0.05	1.08
	SQ	− 0.04	− 1.59	0.11	1.54

#### Analyses for CSM

The hierarchical multiple regression analysis for the CSM score (Table 7) showed a significant model-fit in the first step (*F*(1, 2287) = 313.83, *p* < 0.001, adjusted R^2^ = 0.12, BF_10_ = 1.73 × 10^62^), and a significant positive relationship for age (*β* = 0.35, *t* = 17.72, *p* < 0.001). The inputs of AQ, EQ, and SQ scores significantly changed the explanatory effect of the model (*F*(3, 2286) = 20.16, *p* < 0.001, R^2^ change = 0.02, BF_10_ = 1.89 × 10^9^). This revealed a significant negative relationship for AQ (*β* =  − 0.13, *t* =  − 5.46, *p* < 0.001) and a positive relationship for SQ (*β* = 0.07, *t* = 3.25, *p* = 0.001). These results were consistent for both male and female participants (Supplementary Table 9). The explanatory effect of the model changed significantly in the second step (male: *F*(3, 1144) = 11.92, *p* < 0.001, R^2^ change = 0.03, BF_10_ = 2.34 × 10^4^; female: *F*(3, 1128) = 8.61, *p* < 0.001, R^2^ change = 0.02, BF_10_ = 2.24 × 10^2^), and showed a significant negative relationship for AQ (male: *β* =  − 0.14, *t* =  − 4.21, *p* < 0.001; female: *β* =  − 0.12, *t* =  − 3.51, *p* < 0.001) as well as significant positive relationships for SQ (male: *β* = 0.08, *t* = 2.69, *p* = 0.01; female: *β* = 0.07, *t* = 2.25, *p* = 0.03).

**Table 7:** Results for the multiple regression analysis for CSM in Survey 2.

Model	Variables	*β*	*t*	*p*	VIF
First step	(Intercept)		242.79	< 0.001	
	Age	0.35	17.72	< 0.001	1.00
Second step	(Intercept)		45.67	< 0.001	
	Age	0.31	15.08	< 0.001	1.11
	AQ	− 0.13	− 5.46	< 0.001	1.53
	EQ	0.01	0.32	0.75	1.50
	SQ	0.07	3.25	0.001	1.08

#### Analyses for sex

We tested whether there were differences in the dependent variables (*p* = 0.05/8). As in Survey 1, Mann–Whitney U tests (*p* = 0.05/8) found that AQ and SQ scores were significantly higher for males than for females, and the opposite tendency was found for EQ (Table 8).

**Table 8:** Results for the comparisons between sexes in Survey 2.

Label	W	*p*	BF10
Body temperature	672,299.0	0.02	0.22
AQ	738,803.5	< 0.001	1.15E+02
EQ	509,830.0	< 0.001	4.88E+04
SQ	939,997.5	< 0.001	1.583E+13
CSM	631,916.5	0.26	0.07
Wakeup time	642,438.0	0.64	0.05
Measurement time	627,875.5	0.16	0.13
Time	653,261.5	0.82	0.06

### Analyses for autistic traits

Correlation analyses among autistic traits (*p* = 0.05/3) showed the significant negative correlations between AQ and both EQ (*τ* =  − 0.41, *p* < 0.001, BF_10_ = 4.20 × 10^181^) and SQ (*τ* =  − 0.07, *p* < 0.001, BF_10_ = 1.70 × 10^3^). SQ and EQ were positively correlated (*τ* = 0.12, *p* < 0.001, BF_10_ = 4.06 × 10^15^). These results were consistent in both the male and female participants (Supplementary Table 10).

Partial correlation analyses for autistic traits against age (*p* = 0.05/3) revealed significant negative relationships for AQ (*τ* =  − 0.19, *p* < 0.001) and EQ (*τ* =  − 0.07, *p* < 0.001), and a positive relationship for SQ (*τ* = 0.08, *p* < 0.001). A significant relationship for AQ was consistently observed in both male and female participants, whereas significant relationships for EQ and SQ were found only in males (Supplementary Table 11).

## Discussions

### No significant relationships between autistic traits and body temperature

This study investigated a possible relationship between autistic traits and body temperature by a survey protocol using a large, population-based Japanese sample comprising people in age groups 20s to 70s and an equal distribution of the sexes. Through two surveys, we confirmed that there were no significant relationships between the AQ, EQ, and SQ scores and body temperature measured by axillary thermometers. While we found significant relationships without the female data of particular ages in the first survey, this result was not replicated in the second study. The procedures were identical throughout the two surveys, and the distributions of the dependent variable were very similar between the two surveys performed at different time points. We could consider that the differences in the findings between the two surveys would be incidental, perhaps because of artifacts caused by seasonal differences in atmospheric temperature or other factors. Our findings suggest that ordinary body temperature may not be closely associated with autistic traits. Therefore, our hypothesis that greater autistic traits would be associated with lower body temperatures at the population level, based on the evidence for longitudinal shifts in clinical ASD diagnosis^7^ and body temperatures^16^, was not supported by the current study.

In this study, to measure autistic traits, we used AQ, SQ, and EQ scores based on the idea that ASD-like characteristics are common properties that fall into a continuous distribution among the general population^2–6^. Consistent with previous studies^4,18,19,24^, higher AQ and SQ scores and lower EQ scores were observed for the males compared to the females, and there was a negative relationship between AQ and EQ scores in both surveys. These findings suggest that the measurements of autistic traits were reliable in this study. However, in contrast to previous findings^18,19^, SQ scores were negatively and positively correlated with the AQ and EQ scores, respectively. In additions, some results related to SQ scores were inconsistent between the two surveys. We might consider that the recognition and demands for “systematizing” tendencies in society have been changing with the current times of technology. Further investigations of this matter are beyond the scope of this study.

Consistent with previous findings, we found a significant relationship between body temperature and age^25^ and sex in particular age groups^26^ in both surveys. This indicates that the reported body temperatures were reliable. However, it should be noted that the CSM scores, which are an index of the circadian rhythm reported to affect body temperature^25^, did not have a significant relationship with body temperature. We should note the possibility that participants’ behavior when measuring body temperatures could be irregular, while we excluded the data considered to be outliers in the current study. Also, previous studies on body temperature have mostly used a more precise index, that is, core body temperature. The validity of our findings should be tested in conditions where the body temperature measurements are carefully controlled and/or using core body temperature in future studies. Moreover, previous findings have shown that behavioral characteristics specific to ASD were moderated in children with clinical ASD diagnosis during a fever^17^ and that the decrement in cortical temperature increases brain activity^10–15^ and weakens the contribution of inhibitory inputs to neural activities relative to excitatory inputs^15^. It may be possible that the magnitudes of characteristics specific to autistic traits fluctuate over time at a daily level with changes in body temperature with circadian rhythm^25^. Notably, a recent study using magnetic resonance spectroscopy has also reported that brain temperatures show daily variation as well as differences in brain temperatures across brain regions^39^. It could also be possible that artificial factors such as activity level and metabolism among individuals could affect body and brain temperatures. Future studies should focus on individual changes in ASD characteristics and body and brain temperatures over time.

### Significant relationships between an ASD trait and CSM

We measured the strength of morningness/eveningness with CSM scores as an index of the circadian rhythms and explored possible relationships for autistic traits. Both surveys consistently found a significant negative relationship between CSM and AQ scores for all participants, suggesting that people with higher general autistic traits have more eveningness tendencies. It has been reported that people with clinical ASD diagnosis have circadian rhythm disturbances^29^ and sleep problems^30^. Our findings suggest that these may not be limited to people with clinical ASD diagnosis, but also applicable to the general population with higher autistic traits. Sleep difficulties can be a major cause of mental health problems but are moderated by clinical interventions e.g.^40^. Our findings may contribute to further understanding of difficulties in daily life related to autistic traits, although further investigations are necessary using multiple indices for circadian rhythms and sleep difficulties.

### Significant relationships between age and autistic traits

Both surveys consistently confirmed significant relationships between age and autistic traits for all participants. We consider that the significant negative relationship for AQ is small but valid and worth discussing, whereas the other relationships that show very small relations are not. This result is consistent with a previous finding in the United States^31^. While mixed evidence has been reported for changes in ASD-like characteristics with aging in people with clinical ASD diagnosis^41,42^, our findings suggest that autistic traits could be mitigated with aging in the general population. There are some possible explanations for this finding. The imbalance of excitatory/inhibitory neural processes has been considered one of the potential causes of ASD^8,9,43^. This imbalance could be altered, though it has been suggested that inhibitory processes become weaker with aging^44,45^. Idiosyncratic aspects of sensory and cognitive processes, such as hyper/hyposensitivities and attentional irregularities, are also often reported in individuals with clinical ASD diagnosis^1^. These aspects may change because sensory and cognitive functions generally decline with aging^42^. Furthermore, it may be possible for people to develop strategies to cope with ASD-related characteristics and behaviors with aging^41^. These possibilities should be tested in the future to better understand the malleability of ASD-related characteristics.

## Conclusions

Focusing on the concordance of increment in the number of clinical ASD diagnosis and decrement in body temperature over decades, we tested a possible relationship between the magnitude of autistic traits and body temperature in the general population using a large sample. Through these two surveys, we did not find any significant relationships. In contrast, we consistently found that people with higher AQ scores tended to have a stronger eveningness tendency, and that autistic traits were weaker in older people. Our findings can contribute to understanding the irregularity of circadian rhythms and the malleability of ASD-related characteristics.

## Supplementary Information

Supplementary Information.

## Data Availability

Our data have been made publicly available via the Open Science Framework and can be accessed at https://osf.io/f8bh5/.
