# Supplementary material for: Exploring relationships between autistic traits and body temperature, circadian rhythms, and age
Source: Sci Rep. 2023 Apr 11;13:5888. doi: 10.1038/s41598-023-32449-z (PMC10088634; doi:10.1038/s41598-023-32449-z)
Supplement: Supplementary file 1 — Supplementary Information. [file 41598_2023_32449_MOESM1_ESM.pdf]

Exploring relationships between autistic traits and body temperature, circadian rhythms, and age

Souta Hidaka[1,5*], Mizuho Gotoh[2,3,4], Shinya Yamamoto[2,3], and Makoto Wada[4*]

1. Department of Psychology, Rikkyo University,1-2-26, Kitano, Niiza, Saitama, 352-8558, Japan.
2. Integrative Neuroscience Research Group, Human Informatics and Interaction Research Institute, National Institute of Advanced Industrial Science and Technology (AIST), 1-1-1, Umezono, Tsukuba 305-8568, Japan
3. Graduate School of Comprehensive Human Sciences, University of Tsukuba, 1-1-1, Tennodai, Tsukuba 305-8577, Japan
4. Developmental Disorders Section, Department of Rehabilitation for Brain Functions, Research Institute of National Rehabilitation Center for Persons with Disabilities, 4-1, Namiki, Tokorozawa, Saitama, 359-8555, Japan.
5. Department of Psychology, Faculty of Human Sciences, Sophia University 7-1, Kioi-cho, Chiyoda-ku, Tokyo, 102-8554, Japan

*Corresponding authors:
Souta Hidaka
E-mail: hidaka@sophia.ac.jp
Address: Department of Psychology, Faculty of Human Sciences, Sophia University 7-1, Kioi-cho, Chiyoda-ku, Tokyo, 102-8554, Japan

Makoto Wada
E-mail: wada-makoto@rehab.go.jp
Address: Developmental Disorders Section, Department of Rehabilitation for Brain Functions, Research Institute of National Rehabilitation Center for Persons with Disabilities, 4-1, Namiki, Tokorozawa, Saitama, 359-8555, Japan.

Supplementary figures

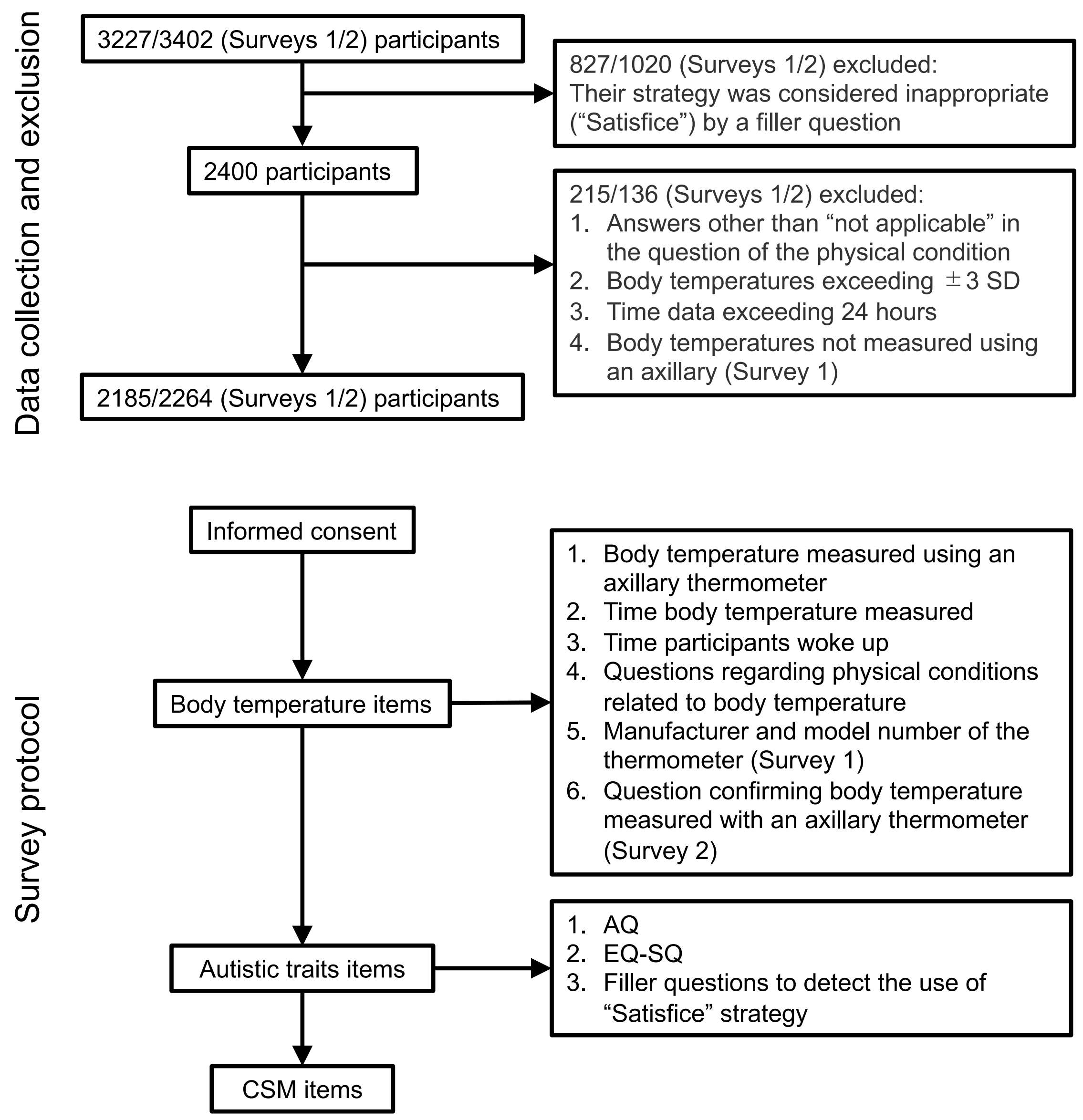

Supplementary figure 1. Flowcharts for data collection and exclusion process and survey protocol.

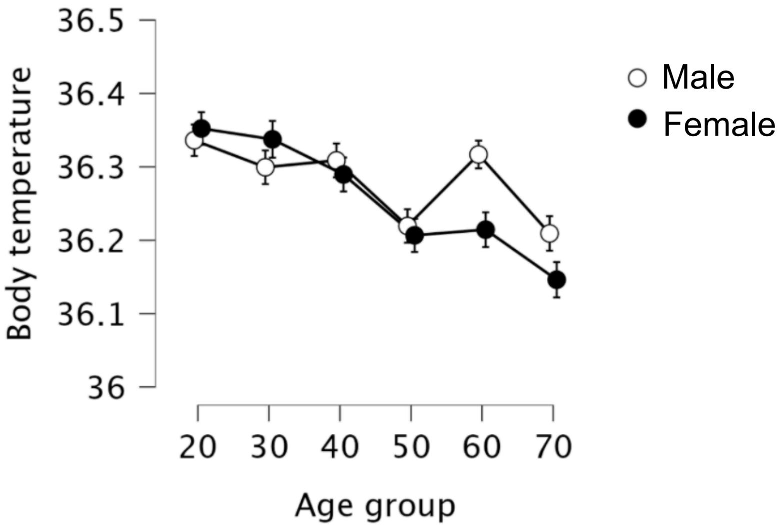

Supplementary figure 2. Data for body temperatures plotted with age group and sex in Survey 1. Error bars represents the standard errors.

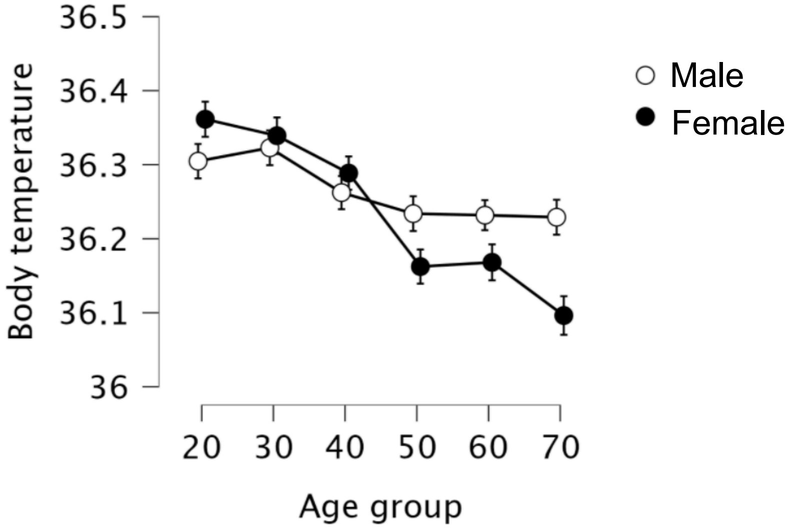

Supplementary figure 3. Data for body temperatures plotted with age group and sex in Survey 2. Error bars represents the standard errors.

Supplementary tables

Supplementary table 1-1. Results for the multiple regression analysis for body temperature for the data of males in Survey 1.

Model	Variables	*β*	*t*	*p*	VIF
First step	(Intercept)		3673.29	< .001	
	Age	−0.08	−2.50	0.01	1.19
	CSM	−0.05	−1.37	0.17	1.19
	Time	−0.01	−0.36	0.72	1.02
	Age × CSM	0.03	1.03	0.31	1.02
	Age × Time	−0.02	−0.55	0.58	1.19
	CSM × Time	−0.02	−0.58	0.56	1.17
Second step	(Intercept)		661.17	< .001	
	Age	−0.10	−2.89	0.00	1.24
	CSM	−0.04	−1.34	0.18	1.22
	Time	−0.01	−0.22	0.83	1.03
	Age × CSM	0.03	0.88	0.38	1.02
	Age × Time	−0.02	−0.57	0.57	1.20
	CSM × Time	−0.02	−0.65	0.51	1.17
	AQ	−0.09	−2.40	0.02	1.65
	EQ	−0.09	−2.28	0.02	1.81
	SQ	−0.04	−1.31	0.19	1.25

Supplementary table 1-2. Results for the multiple regression analysis for body temperature for the data of females in Survey 1.

Model	Variables	*β*	*t*	*p*	VIF
First step	(Intercept)		3131.17	< .001	
	Age	−0.19	−5.43	< .001	1.15
	CSM	0.05	1.39	0.17	1.29
	Time	0.03	0.86	0.39	1.19
	Age × CSM	0.00	−0.02	0.98	1.21
	Age × Time	−0.03	−0.84	0.40	1.29
	CSM × Time	−0.02	−0.58	0.57	1.12
Second step	(Intercept)		622.29	< .001	
	Age	−0.20	−5.55	< .001	1.19
	CSM	0.05	1.26	0.21	1.30
	Time	0.03	0.80	0.43	1.20
	Age × CSM	0.00	0.06	0.95	1.21
	Age × Time	−0.03	−0.85	0.39	1.29
	CSM × Time	−0.02	−0.57	0.57	1.12
	AQ	−0.04	−1.08	0.28	1.48
	EQ	0.00	0.06	0.96	1.47
	SQ	0.01	0.36	0.72	1.07

Supplementary table 2. Results for the multiple regression analysis for body temperature excluding females' 60s and 70s data in Survey 1.

Model	Variables	*β*	*t*	*p*	VIF
First step	(Intercept)		4603.96	< .001	
	Age	−0.11	−4.54	< .001	1.15
	CSM	−0.02	−0.57	0.57	1.27
	Time	0.01	0.42	0.67	1.03
	Age × CSM	0.00	0.17	0.87	1.12
	Age × Time	−0.03	−1.16	0.24	1.19
	CSM × Time	−0.02	−0.79	0.43	1.17
Second step	(Intercept)		874.99	< .001	
	Age	−0.12	−4.78	< .001	1.23
	CSM	−0.02	−0.73	0.46	1.30
	Time	0.01	0.54	0.59	1.03
	Age × CSM	0.00	0.08	0.94	1.13
	Age × Time	−0.03	−1.19	0.23	1.19
	CSM × Time	−0.02	−0.80	0.43	1.17
	AQ	−0.09	−2.93	0.00	1.55
	EQ	−0.07	−2.35	0.02	1.60
	SQ	−0.03	−1.06	0.29	1.13

Supplementary table 3. Results for the multiple regression analysis for body temperature excluding two manufactures' data in Survey 1.

ANOVA	Adjusted R2	*F*	R2 change	*p*
First step	0.02	7.59		< .001
Second step	0.02	1.16	0.00	0.33

Model	Variables	*β*	*t*	*p*	VIF
First step	(Intercept)		4493.02	< .001	
	Age	−0.16	−6.08	< .001	1.20
	CSM	0.02	0.65	0.52	1.23
	Time	0.00	0.08	0.94	1.01
	Age × CSM	0.03	1.07	0.29	1.03
	Age × Time	−0.03	−0.96	0.34	1.20
	CSM × Time	−0.03	−0.98	0.33	1.20
Second step	(Intercept)		860.79	< .001	
	Age	−0.17	−6.26	< .001	1.27
	CSM	0.02	0.57	0.57	1.26
	Time	0.00	0.13	0.90	1.01
	Age × CSM	0.03	1.02	0.31	1.04
	Age × Time	−0.02	−0.93	0.35	1.20
	CSM × Time	−0.03	−0.98	0.33	1.21
	AQ	−0.05	−1.56	0.12	1.57
	EQ	−0.05	−1.60	0.11	1.56
	SQ	0.00	−0.15	0.88	1.09

Supplementary table 4-1. Results for the multiple regression analysis for CSM for the data of males in Survey 1.

Model	Variables	*β*	*t*	*p*	VIF
First step	(Intercept)		167.55	< .001	
	Age	0.39	14.30	< .001	1.00
Second step	(Intercept)		29.76	< .001	
	Age	0.36	12.87	< .001	1.08
	AQ	−0.12	−3.32	< .001	1.62
	EQ	−0.01	−0.19	0.85	1.82
	SQ	0.08	2.47	0.01	1.25

Supplementary table 4-2. Results for the multiple regression analysis for CSM for the data of females in Survey 1.

Model	Variables	*β*	*t*	*p*	VIF
First step	(Intercept)		171.51	< .001	
	Age	0.40	14.20	< .001	1.00
Second step	(Intercept)		32.68	< .001	
	Age	0.36	12.33	< .001	1.12
	AQ	−0.11	−3.20	0.001	1.54
	EQ	0.03	0.73	0.46	1.50
	SQ	0.01	0.24	0.81	1.09

Supplementary table 5. Results for the correlation analyses among ASD traits for male and the data of females in Survey 1.

Data	pair	*τ*	*p*	BF
Male	AQ-EQ	−0.45	< .001	1.30E+107
	AQ-SQ	−0.19	< .001	9.74E+18
	EQ-SQ	0.30	< .001	3.45E+46
Female	AQ-EQ	−0.40	< .001	1.81E+85
	AQ-SQ	−0.10	< .001	2.57E+03
	EQ-SQ	0.16	< .001	7.10E+12

Supplementary table 6. Results for the correlation analyses between age and each ASD traits for male and the data of females in Survey 1.

Data	variable	*τ*	*p*
Male	AQ	−0.14	< .001
	EQ	−0.07	0.001
	SQ	0.06	0.003
Female	AQ	−0.21	< .001
	EQ	−0.03	0.11
	SQ	0.01	0.56

Supplementary table 7-1. Results for the multiple regression analysis for body temperature for the data of males in Survey 2.

Model	Variables	*β*	*t*	*p*	VIF
First step	(Intercept)		3627.38	< .001	
	Age	−0.10	−3.14	0.00	1.17
	CSM	0.02	0.65	0.51	1.16
	Time	0.05	1.64	0.10	1.04
	Age × CSM	−0.02	−0.65	0.51	1.03
	Age × Time	−0.02	−0.58	0.56	1.11
	CSM × Time	0.03	0.91	0.36	1.12
Second step	(Intercept)		657.37	< .001	
	Age	−0.12	−3.49	< .001	1.25
	CSM	0.01	0.37	0.71	1.20
	Time	0.05	1.60	0.11	1.05
	Age × CSM	−0.02	−0.65	0.52	1.03
	Age × Time	−0.02	−0.68	0.50	1.11
	CSM × Time	0.03	0.89	0.38	1.13
	AQ	−0.04	−1.14	0.25	1.55
	EQ	−0.04	−1.10	0.27	1.61
	SQ	0.05	1.65	0.10	1.20

Supplementary table 7-2. Results for the multiple regression analysis for body temperature for the data of females in Survey 2.

Model	Variables	*β*	*t*	*p*	VIF
First step	(Intercept)		3430.09	< .001	
	Age	−0.25	−7.99	< .001	1.16
	CSM	−0.07	−2.28	0.02	1.14
	Time	−0.02	−0.62	0.53	1.03
	Age × CSM	−0.02	−0.64	0.52	1.03
	Age × Time	0.00	−0.01	0.99	1.11
	CSM × Time	0.00	−0.04	0.97	1.11
Second step	(Intercept)		658.56	< .001	
	Age	−0.26	−7.95	< .001	1.25
	CSM	−0.07	−2.28	0.02	1.17
	Time	−0.02	−0.70	0.49	1.03
	Age × CSM	−0.02	−0.60	0.55	1.03
	Age × Time	0.00	−0.05	0.96	1.11
	CSM × Time	0.00	0.00	1.00	1.11
	AQ	−0.04	−1.01	0.31	1.52
	EQ	−0.02	−0.44	0.66	1.51
	SQ	−0.02	−0.70	0.49	1.14

Supplementary table 8. Results for the multiple regression analysis for body temperature excluding females' 50s and 70s data in Survey 2.

Model	Variables	*β*	*t*	*p*	VIF
First step	(Intercept)		4582.74	< .001	
	Age	−0.14	−5.82	< .001	1.14
	CSM	−0.02	−0.80	0.42	1.18
	Time	0.01	0.40	0.69	1.06
	Age × CSM	−0.01	−0.50	0.62	1.04
	Age × Time	−0.01	−0.50	0.62	1.15
	CSM × Time	0.01	0.42	0.68	1.11
Second step	(Intercept)		867.95	< .001	
	Age	−0.16	−6.26	< .001	1.23
	CSM	−0.03	−1.12	0.26	1.21
	Time	0.01	0.39	0.70	1.06
	Age × CSM	−0.01	−0.55	0.59	1.05
	Age × Time	−0.01	−0.47	0.64	1.15
	CSM × Time	0.01	0.41	0.68	1.11
	AQ	−0.06	−2.23	0.03	1.51
	EQ	−0.04	−1.35	0.18	1.50
	SQ	0.03	1.18	0.24	1.11

Supplementary table 9-1. Results for the multiple regression analysis for CSM for the data of males in Survey 2.

Model	Variables	β	t	p	VIF
First step	(Intercept)		41.55	< .001	
	Age	0.36	13.04	< .001	1.00
Second step	(Intercept)		20.70	< .001	
	Age	0.32	11.02	< .001	1.11
	AQ	−0.14	−4.21	< .001	1.52
	EQ	−0.01	−0.37	0.71	1.61
	SQ	0.08	2.69	0.01	1.18

Supplementary table 9-2. Results for the multiple regression analysis for CSM for the data of females in Survey 2.

Model	Variables	β	t	p	VIF
First step	(Intercept)		43.43	< .001	
	Age	0.34	11.98	< .001	1.00
Second step	(Intercept)		21.44	< .001	
	Age	0.30	10.13	< .001	1.12
	AQ	−0.12	−3.52	< .001	1.51
	EQ	0.01	0.21	0.83	1.53
	SQ	0.07	2.25	0.03	1.13

Supplementary table 10. Results for the correlation analyses among ASD traits for male and the data of females in Survey 2.

Data	pair	τ	*p*	BF
Male	AQ-EQ	−0.41	< .001	4.72E+92
	AQ-SQ	−0.16	< .001	7.43E+12
	EQ-SQ	0.22	< .001	3.20E+25
Female	AQ-EQ	−0.51	< .001	1.80E+71
	AQ-SQ	−0.09	0.004	3.019
	EQ-SQ	0.32	< .001	3.92E+24

Supplementary table 11. Results for the correlation analyses between age and each ASD traits for male and the data of females in Survey 2.

Data	variable	τ	*p*
Male	AQ	−0.17	< .001
	EQ	−0.09	< .001
	SQ	0.09	< .001
Female	AQ	−0.20	< .001
	EQ	−0.05	0.02
	SQ	0.03	0.18
